# Insights into the Molecular Regulation of Lignin Content in Triploid Poplar Leaves

**DOI:** 10.3390/ijms23094603

**Published:** 2022-04-21

**Authors:** Tingting Xu, Shuwen Zhang, Kang Du, Jun Yang, Xiangyang Kang

**Affiliations:** 1 National Engineering Research Center of Tree Breeding and Ecological Remediation, Beijing Forestry University, Beijing 100083, China; tingtingxu0411@163.com (T.X.); shuwenzhang@bjfu.edu.cn (S.Z.); dukang@bjfu.edu.cn (K.D.); 2 Key Laboratory of Genetics and Breeding in Forest Trees and Ornamental Plants, Ministry of Education, College of Biological Sciences and Technology, Beijing Forestry University, Beijing 100083, China

**Keywords:** triploid, lignin content, gene expression, regulatory mechanism

## Abstract

After polyploidization, plants usually undergo some morphological and physiological changes, including the lignin content of polyploids usually becoming lower than that of diploids. However, the regulatory mechanism of the variation of lignin content in polyploid plants remains unclear. Therefore, in this research, we used full-sib poplar triploids and diploids to explore the molecular regulatory basis of lignin content in poplar triploid leaves through the determination of lignin content, the observation of xylem cells, and transcriptome sequencing. The results showed that the lignin content of triploid leaves was significantly lower than that of diploid leaves. The xylem cells of triploid leaves were significantly larger than those of diploids. Transcriptome sequencing data show that most lignin biosynthesis genes were significantly downregulated, and genes related to cell growth were mostly upregulated in triploid leaves compared with diploid leaves. In addition, co-expression network analysis showed that several transcription factors might be involved in the regulation of lignin biosynthesis. Consequently, the altered expression of genes related to lignin might lead to the reduced lignin content in triploids. These results provide a theoretical basis for further exploring the molecular mechanism of the variation of polyploid lignin content and the utilization of polyploid lignocellulosic resources.

## 1. Introduction

Lignin is a major component of the secondary cell wall that is deposited when cell differentiation is completed and the secondary cell wall starts thickening [1]. Lignin is also the second most abundant plant lignocellulosic material in nature [2]. Lignin plays an important role in mechanical support, water transportation, and stress defense during plant growth and development [3]. However, it interweaves with cellulose and hemicellulose in the secondary cell wall, which seriously affects the depolymerization and utilization of plant lignocellulosic resources [4]. The high lignin content in plants is the main restrictive factor for lignocellulosic bioenergy production, pulp and paper making, and forage digestion [4,5,6]. Therefore, plants with properly low lignin content can better meet the needs for human social production practices.

Plant polyploidy, accompanied by an increase in chromosome numbers, usually brings about some morphological and physiological changes through affecting the various physiological activities and metabolic processes of plants [7,8]. For example, plant polyploidy usually improves the growth rate, wood quality, and stress resistance [4,9,10,11]. Studies have shown that the cell-wall components of polyploid plants tend to be altered. In *Populus tomentosa*, compared with diploids, triploids were characterized by longer and wider fiber cells, lower lignin content, which decreased by 17.9%, and higher cellulose content; therefore, they were easier to cook and soften in pulp and paper industrial processes, and energy consumption was reduced [12]. In triploid shrub willows, the lignin content significantly decreased, and the lignin monomer composition also changed [13]. Compared with *Arabidopsis* diploids, the lignin contents of tetraploids, hexaploids, and octaploids were decreased by 20%, 50%, and 55%, respectively [14]. A similar phenomenon was found for polyploid crops such as rice and potatoes; the lignin content of polyploids was significantly lower than that of diploids [15,16].

With the development of high-throughput sequencing technology, an increasing number of studies have led to in-depth discussions about the gene expression and regulatory mechanisms of polyploid plants. For example, in poplar triploids, the expression of genes related to vegetative growth has a dose effect, which enhances the capacities for photosynthesis, carbon fixation, and sucrose and starch metabolism, accompanied by a higher chlorophyll content and lower chlorophyll degradation, delaying leaf senescence [17]. Due to the gene dosage effect of polyploidy, some plant physiological and biochemical processes are strengthened along with more vigorous metabolism; consequently, the contents of biochemical components are also increased. However, in contrast to the higher photosynthesis, carbon fixation, and sucrose and starch metabolism, lignin content was lower in poplar triploids than in diploids. Why does the lignin content decrease in triploids? The mechanism behind the variation remains unclear. The analysis of the regulation in leaf lignin biosynthesis is beneficial to the development and utilization of polyploid-based leaf bioenergy. The existing studies on stems could not completely characterize the mechanism of lignin biosynthesis in leaves. The development and utilization of leaf-based biomass energy or important secondary products in polyploids still requires a more accurate regulatory mechanism model.

Therefore, in this study, we used full-sib poplar triploids and diploids as study materials to explore the molecular mechanism of the regulation of lignin content in poplar triploids by determining the lignin content, observing xylem cells and performing transcription sequencing analysis. These results provide a theoretical basis for further exploring the molecular mechanism of lignin trait variation in other polyploid plants or tissues, e.g., woody tissues, and the utilization of polyploid lignocellulosic resources. Moreover, it can also enrich the theories on the molecular mechanisms behind the formation of advantageous traits of poplar triploids.

## 2. Results

### 2.1. Analysis of Lignin Content in Triploid and Diploid Leaves

**Figure 1:**
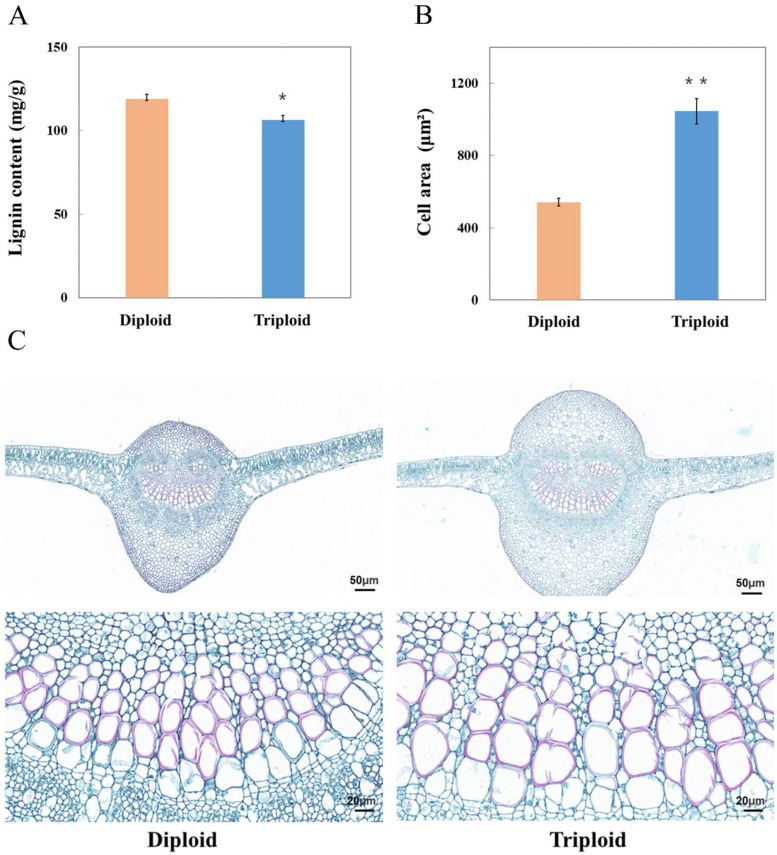
Analysis of lignin content in triploid and diploid leaves. (**A**) Lignin content in triploid and diploid leaves. (**B**) Cell area measurement of triploid and diploid xylem vessels. (**C**) The observation of xylem cells in triploid and diploid leaves. Values are the means ± SE of three independent experiments. The asterisk indicates significant differences (* *p* < 0.05, ** *p* < 0.01). Bar = 50/20 µm.

The fifth leaf was in the growth stage, with its net photosynthetic rate and leaf area increasing. However, the net photosynthetic rate and leaf area peaked at the tenth leaf, meaning that the cell growth and leaf function tended to be stable [18]. In this study, juvenile and mature leaves represented the fifth and tenth leaves, respectively. In order to compare the lignin contents of different ploidy plants, the fifth leaves of triploids and diploids were used to determine the lignin content. The results show that the lignin content in triploid leaves was significantly lower than that in diploid leaves (Figure 1A). Lignin is mainly deposited in specific vascular tissue cells. Thus, we further observed the xylem cell microstructure of the main veins by histochemical staining. The results show that the xylem vessels of triploid leaves were significantly larger than those of diploid leaves (Figure 1B,C).

### 2.2. Analysis of Transcriptome Sequencing Data

To analyze the gene expression differences between triploid and diploid poplar leaves, transcriptome sequencing was performed on juvenile and mature leaves of different ploidy plants. A total of 12 cDNA libraries were constructed, including three biological replicates. In total, 90.73%, 90.43%, 87.50%, and 89.27% of the average reads were matched to genomic locations, and uniquely mapped reads accounted for 84.90%, 84.13%, 82.13%, and 84.07% in the Dip_JL, Tri_JL, Dip_ML, and Tri_ML libraries, respectively (Table S2).

**Figure 2:**
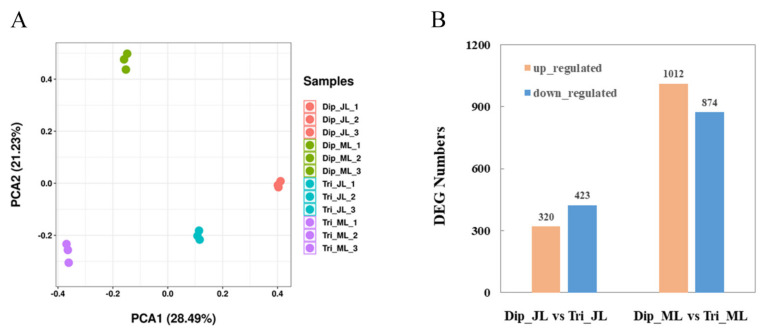
Analysis of transcriptome sequencing data. (**A**) Principal component analysis (PCA) analysis of transcriptome sequencing samples. (**B**) Numbers of differentially expressed upregulated and downregulated genes in juvenile and mature leaves between triploids and diploids. Dip_JL, juvenile diploid leaves; Dip_ML, mature diploid leaves; Tri_JL, juvenile triploid leaves; Tri_ML, mature triploid leaves.

To assess the reliability of the tested samples, principal component analysis (PCA) was performed (Figure 2A). The results show that there was a high degree of similarity among the biological replicates of each sample, and that different ploidy plants and different leaves had different gene expression patterns, indicating that the sequencing data were relatively reliable and suitable for further analysis. In this study, the screening threshold for differentially expressed genes (DEGs) was |FC| ≥ 2 and FDR < 0.05. Compared with diploids, there were 743 DEGs, including 320 upregulated and 423 downregulated genes in juvenile leaves (Figure 2B). A total of 1886 DEGs were identified in mature leaves; 1012 were upregulated, and 874 were downregulated (Figure 2B).

### 2.3. Functional Enrichment Analysis of DEGs

**Figure 3:**
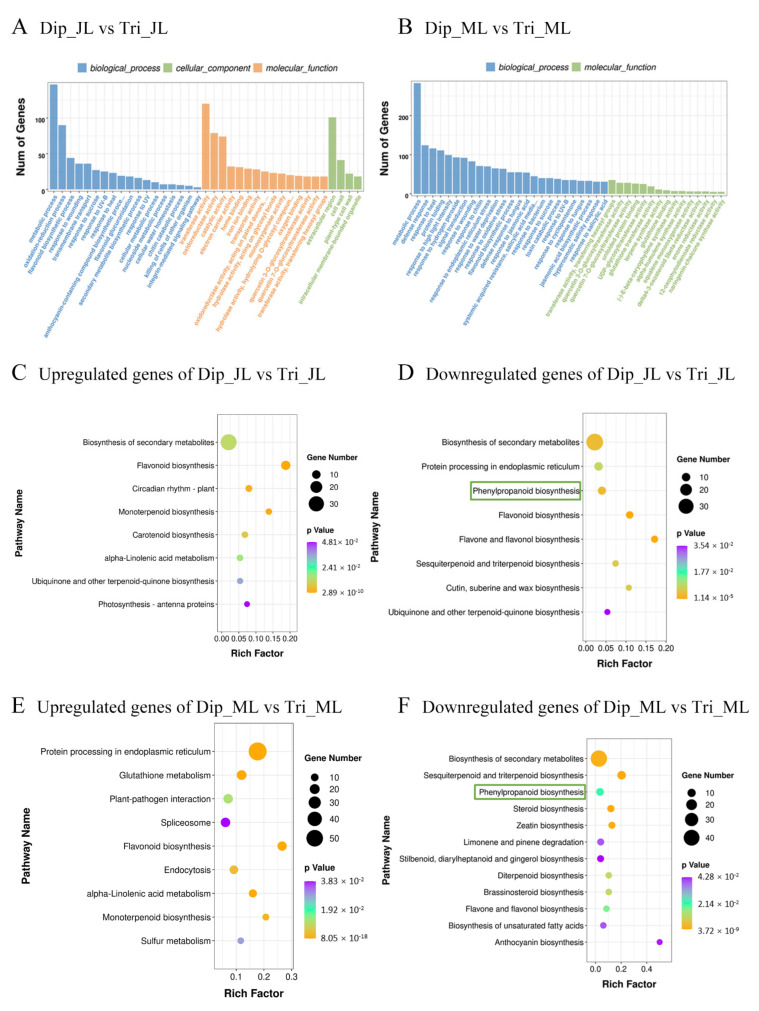
Functional enrichment analysis of differentially expressed genes (DEGs). (**A**) Gene Ontology (GO) analysis of DEGs between juvenile triploid and diploid leaves. (**B**) GO analysis of DEGs between mature triploid and diploid leaves. (**C**) Kyoto Encyclopedia of Genes and Genomes (KEGG) analysis of differentially upregulated genes between juvenile triploid and diploid leaves. (**D**) KEGG analysis of differentially downregulated genes between triploid and diploid mature leaves. (**E**) KEGG analysis of differentially upregulated genes between mature triploid and diploid leaves. (**F**) KEGG analysis of differentially downregulated genes between mature triploid and diploid leaves. The *Y*-axis indicates the KEGG pathway; the *X*-axis indicates the rich factor. The dot size indicates the number of DEGs of the pathway, and the dot color indicates the *p*-value. Dip_JL, juvenile diploid leaves; Dip_ML, mature diploid leaves; Tri_JL, juvenile triploid leaves; Tri_ML, mature triploid leaves.

To characterize the biological roles of DEGs, Gene Ontology (GO) enrichment analysis was performed at three levels: biological process (BP), molecular function (MF), and cellular component (CC) (Figure 3A,B; Table S3). The major GO items are shown in Figure 3A,B (FDR < 0.05). In juvenile leaves, the DEGs were significantly enriched for 18 BP items, mainly including “metabolic process”, “oxidation–reduction process”, and “flavonoid biosynthesis process”. The DEGs were significantly enriched for 45 MF items, the most significant being “transferase activity”, “oxidation–reduction enzyme activity”, and “catalytic activity”. Four items were enriched for CC items, among which “extracellular region” and “cell wall” were the most significant. In mature leaves, the DEGs were significantly enriched for 53 BP items, primarily including “metabolic process”, “defense stress response”, “heat response”, and “protein folding”. A total of 15 MF items were enriched, the most significant being “transferase activity”.

To further analyze the specific metabolic pathways of the DEGs involved, we performed Kyoto Encyclopedia of Genes and Genomes (KEGG) pathway enrichment analysis (Figure 3C–F; Table S3). All the significantly enriched KEGG pathways (*p* < 0.05) are shown in Figure 3C–F. In juvenile leaves, the upregulated genes were mainly enriched for “biosynthesis of secondary metabolites”, “flavonoid biosynthesis”, and “circadian rhythm—plant” (Figure 3C). The downregulated genes were mainly enriched for “the biosynthesis of secondary metabolites”, “protein processing in endoplasmic reticulum”, and “phenylpropanoid biosynthesis” (Figure 3D). In mature leaves, the pathways of “protein processing in endoplasmic reticulum”, “glutathione metabolism”, and “plant–pathogen interaction” were mainly enriched in upregulated genes (Figure 3E). The pathways of “biosynthesis of secondary metabolites”, “sesquiterpenoid and triterpenoid biosynthesis”, and “phenylpropanoid biosynthesis” were mainly enriched in differentially downregulated genes (Figure 3F). Noteworthily, the downregulated genes were all significantly enriched in phenylpropanoid biosynthesis pathways in both leaves.

In conclusion, the enrichment results show that the DEGs in juvenile triploid leaves were mainly involved in the processes of cell growth, such as metabolite synthesis, physiological enzyme activities, and cell-wall activities. However, mature leaves gradually participated in the processes of various environmental interactions such as stimulus responses and defense stresses.

### 2.4. Profiling of Differentially Expressed Lignin Biosynthesis Genes

Lignin is synthesized through the phenylpropanoid biosynthesis pathway, with the participation of many enzymes and genes. The lignin biosynthesis genes are mainly expressed during secondary cell-wall biosynthesis and eventually lead to lignin deposition in the cell wall [1]. In our current study, cell growth was relatively stable in mature leaves, while cell growth, including cell-wall activities, was ongoing in juvenile leaves.

**Figure 4:**
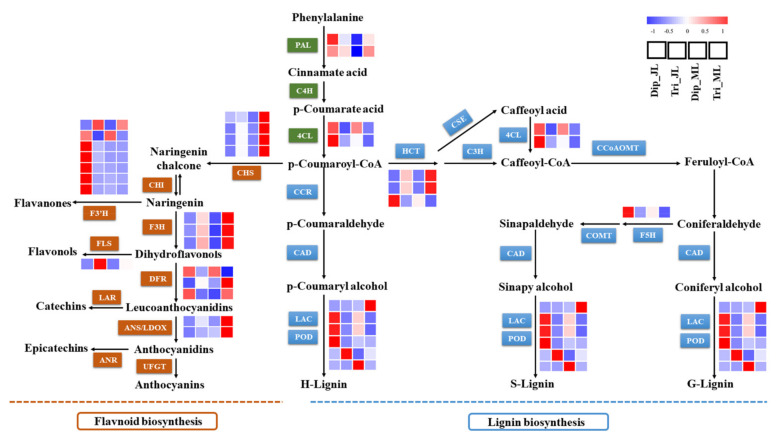
Expression profiles of DEGs involved in the lignin biosynthesis and flavonoid biosynthesis. The color scale represents the log−transformed RPKM value. PAL, phenylalanine ammonia-lyase; C4H, cinnamate 4−hydroxylase; 4CL, 4−coumarate:CoA ligase; C3H, *p*−coumarate 3−hydroxylase; HCT, *p*−hydroxycinnamoyl−CoA:quinate/shikimate *p*−hydroxycinnamoyltransferase; CSE, caffeoyl shikimate esterase; CCoAOMT, caffeoyl−CoA *O*−methyltransferase; COMT, caffeic acid *O*-methyltransferase; F5H, ferulate 5−hydroxylase; CCR, cinnamoyl−CoA reductase; CAD, cinnamyl alcohol dehydrogenase; LAC, laccase; POD, peroxidase; CHS, chalcone synthase; CHI, chalcone isomerase; F3H, flavanone 3−hydroxylase; DFR, dihydroflavonol 4−reductase; ANS/LDOX, anthocyanidin synthase/leucoanthocyanidin dioxygenase; F3′H, flavonol synthase; FLS, flavonol synthase; LAR, leucoanthocyanidin reductase; ANR, anthocyanidin reductase; UFGT, UDP glucose−flavonoid 3−*o*−glycosyltranferase. Dip_JL, juvenile diploid leaves; Dip_ML, mature diploid leaves; Tri_JL, juvenile triploid leaves; Tri_ML, mature triploid leaves.

Compared with diploids, a total of eight and 11 DEGs related to lignin biosynthesis were observed in triploid juvenile and mature leaves, respectively (Figure 4; Table S4). Among them, there were five common genes differentially expressed in both leaves. PAL (phenylalanine ammonia lyase) is the first rate-limiting enzyme in this pathway. Two *PAL1* genes were upregulated in mature triploid leaves compared with diploid leaves. A key enzyme that catalyzes the formation of CoA esters is 4CL (4-coumaroyl: CoA ligase). Two *4CL2* genes were downregulated in both triploid leaves compared with diploid leaves. HCT (hydroxycinnamoyl-CoA shikimate) is also a key enzyme in lignin biosynthesis. In triploids, there was one *HCT* gene downregulated in juvenile leaves and two *HCTs* upregulated in mature leaves compared with diploids. F5H (ferulic acid 5-hydroxylase) is necessary for lignin monomer synthesis. *FAH1* was downregulated in both triploid leaves compared with diploids. In addition, PODs (peroxidases) play a role in the oxidative polymerization of lignin monomers at the end of this pathway. Compared with diploids, there were four *PODs* differentially expressed, three of which were downregulated in juvenile triploid leaves. There were also four *PODs* differentially expressed in mature triploid leaves, two of which were downregulated. A shared *POD* (Potri.006G129900) was downregulated in both triploid leaves. These results indicate that most lignin biosynthesis genes were downregulated in triploids, especially in juvenile leaves, which might be related to the decrease in lignin content.

### 2.5. Profiling of DEGs Related to Cell Growth

**Figure 5:**
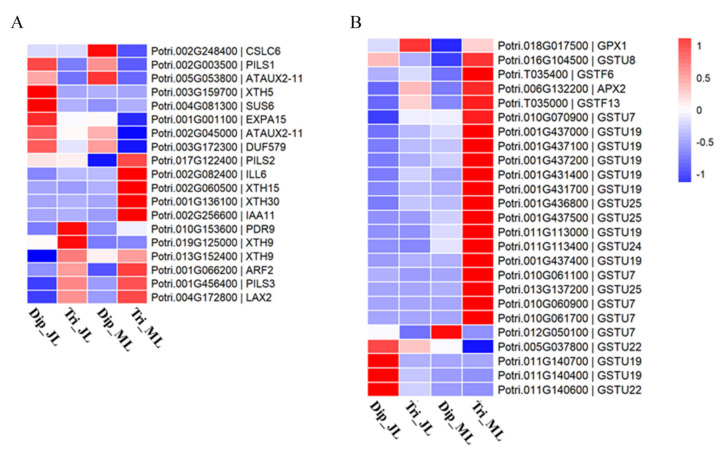
Expression profiles of DEGs. (**A**) DEGs related to cell growth. (**B**) DEGs related to antioxidant enzymes. The color scale represents the log−transformed RPKM value. Dip_JL, juvenile diploid leaves; Dip_ML, mature diploid leaves; Tri_JL, juvenile triploid leaves; Tri_ML, mature triploid leaves.

In our research, we found that the xylem cells in triploid leaves were significantly larger than those in diploid leaves. Therefore, we further analyzed the expression pattern of genes related to cell growth, and some DEGs between triploid and diploid leaves were found (Figure 5A; Table S4).

*XTHs* (xyloglucan endotransglucosylase/hydrolases) are key genes related to cell-wall remodeling. In this study, a total of five *XTHs* were significantly differentially expressed in triploids compared with diploids, most of which were upregulated. Two *XTH9* genes were upregulated in juvenile leaves, whereas *XTH15* and *XTH30* were upregulated in mature leaves. Expansins are also important regulators involved in cell expansion. In this study, most genes encoding expansins were not differentially expressed, but *EXPA15* was differentially downregulated in mature triploid leaves compared with diploid. Phytohormones are also important for cell expansion during plant growth and development. In this study, we found that some auxin-related genes were differentially expressed between triploids and diploids. Genes associated with auxin transportation were mostly upregulated in triploid leaves compared with diploid leaves, such as *PISL*, *LAX2*, and *PDR9*. Three auxin signal transduction inhibitors *AUX2–11* were downregulated in both triploid leaves compared with diploid leaves. Genes related to auxin responses, such as *ARF2*, *IAA11*, and *ILL6*, also showed higher expression in mature triploid leaves than in diploid leaves.

In addition, as the other main cell-wall components, cellulose- and hemicellulose-related genes were also analyzed in this study. The results show that only *SUS6* (sucrose synthase 6) was differentially downregulated in the cellulose biosynthesis of triploid leaves compared with diploids, while the *CESAs* (cellulose synthase), which were crucial for cellulose biosynthesis, were not differentially expressed. Only *DUF57* and *CSCL6* (cellulose synthase-like C6) were differentially downregulated in the hemicellulose biosynthesis of triploid mature leaves compared with diploid, while *IRXs* (irregular xylem), which are important for xylan synthesis, were not differentially expressed.

Consequently, *XTHs* and auxin-related genes might be the major regulators to promote the cell growth in triploid leaves compared with diploids.

### 2.6. Profiling of DEGs Related to Plant Stress Resistance

Plant polyploidy usually results in stronger resistance to various stresses compared to diploidy. Lignin accumulation is an effective pathway for plant stress resistance. However, in our study, triploids had a lower lignin content than diploids; thus, we speculated that there may be some other pathways for plant stress resistance in triploids.

We found that a few antioxidant enzyme genes associated with stress resistance were differentially expressed (Figure 5B; Table S4). *APX2* is a gene encoding ascorbate peroxidase, which was upregulated in triploids compared with diploids. There were eight *GSTs* related to glutathione metabolism that were differentially expressed in juvenile triploid leaves, and half of them were upregulated. In mature triploid leaves, 20 *GSTs* were differentially expressed, and 18 *GSTs* were upregulated, indicating that mature leaves were more active in response to stress defense than juvenile leaves.

In addition, we also found that some DEGs involved in flavonoid biosynthesis shared the phenylpropanoid metabolic pathway with lignin biosynthesis, and most of them had higher expression in triploids than in diploids (Figure 4; Table S4). Flavonoids play an important role in defending against stresses and promoting growth and development in plants. CHS (chalcone synthase) is a key enzyme involved in the biosynthesis of flavonoids. In this study, there were three *CHSs* and one *CHS* gene that were upregulated in juvenile and mature leaves, respectively. F3H encodes flavanone 3-hydroxylase and regulates flavonoid biosynthesis. There were three *F3Hs* gene differentially expressed in triploids compared with diploids, one of which was upregulated in juvenile leaves and two of which were upregulated in mature leaves. F3′H encodes flavonoid 3′ hydroxylase, related to flavonoid biosynthesis. Compared with diploids, seven *F3′H* and two *F3′H* genes were differentially expressed in juvenile and mature triploid leaves, respectively, and most of the *F3′Hs* were downregulated in juvenile triploid leaves. *FLS1* encodes a flavonol synthase involved in the synthesis of flavonols, which was differentially upregulated in both triploid leaves. *LDOX* encodes a leucoanthocyanidin dioxygenase involved in proanthocyanin biosynthesis, which was upregulated in both triploid leaves. *DFR* encodes dihydroflavonol reductase, involved in the biosynthesis of anthocyanins. There were three *DFRs* differentially expressed in mature triploid leaves, two of which were upregulated.

These results show that some other DEGs related to stress resistance were mostly upregulated in triploids compared with diploids, indicating that triploids may gain stronger resistance through other pathways.

### 2.7. Analysis of Differentially Expressed Transcription Factors

**Figure 6:**
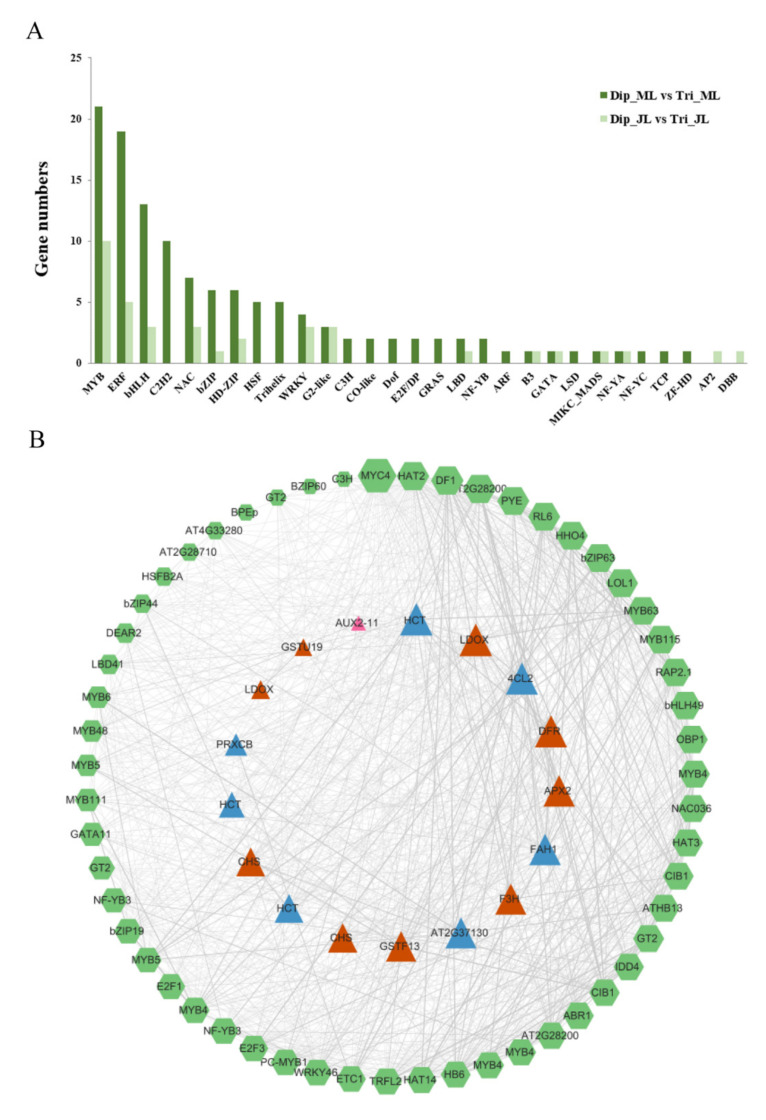
Analysis of differentially expressed transcription factors (TFs). (**A**) The distribution of differentially expressed TFs in juvenile and mature leaves. (**B**) Co-expression network between structural genes (SGs) and TFs. Green hexagonal nodes represent TFs. Triangular nodes represent SGs, of which blue nodes are lignin structural genes, brown nodes are genes related to stress resistance, and pink nodes are genes related to cell growth. The node size is positively correlated with the gene degree. The width of the connecting line is positively related to the correlation between genes. Dip_JL, juvenile diploid leaves; Dip_ML, mature diploid leaves; Tri_JL, juvenile triploid leaves; Tri_ML, mature triploid leaves.

Transcription factors (TFs) play crucial roles in regulating lignin biosynthesis. In this study, 38 differentially expressed TFs were identified in juvenile leaves, composed of 17 TF families. A total of 122 differentially expressed TFs involving 28 TF families were identified in mature leaves. The overall distribution patterns of the TFs are shown in Figure 6, most of which belonged to TF families such as MYB, ERF, NAC, and bHLH (Figure 6A; Table S5).

To identify TFs significantly associated with lignin synthesis, we constructed a co-expression network using DEGs related to lignin including 15 lignin biosynthesis structural genes, nine genes related to cell growth, 11 genes related to stress resistance, and all the differentially expressed TFs (Figure 6B; Table S5). The screening threshold for high correlation was *r* > 0.85 and *p* < 0.05. The larger nodes have stronger connectivity degrees, indicating that the genes may be more important. In this co-expression network, we identified 162 pairs correlated between 14 lignin structural genes and 53 TFs. Among the lignin structural genes, *HCT* (Potri.001G042900), 4*CL2* (Potri.003G188500), *FAH1* (Potri.007G016400), and *POD* (Potri.006G129900) directly connected with the greatest numbers of TFs, all downregulated in triploids compared with diploids. We further analyzed TFs directly linked to lignin structural genes in the network, and the results show that 50 TFs came from 20 families, among which the MYB family had the largest number, with 14 TFs. In addition, the network showed that TFs with high degrees of connectivity were mainly involved in the processes of phenylpropanoid biosynthesis, cell growth, leaf development, and stress response, and they were mainly composed of MYB and bHLH family members (Table S5). Among them, *MYB63* is a transcriptional activator of lignin biosynthesis. *MYB63* connected with the largest numbers of lignin structural genes, and its expression was lower in mature triploid leaves than in diploid leaves. We also found that *MYB115*, associated with proanthocyanidin biosynthesis, was upregulated in both juvenile and mature triploid leaves compared with diploid leaves, which may play an important role in the regulation of flavonoids synthesis. *MYC4*, a bHLH transcription factor, could participate in the regulation of secondary cell-wall synthesis induced by blue light [19]. It was differentially upregulated in triploids with low expression and may work under the induction of blue light. *MYB4*, a repressor involved in the phenylpropanoid pathway, was differentially downregulated in mature triploid leaves compared with diploids. Other TFs have not yet been reported to be involved in lignin synthesis; whether they can directly or indirectly participate in lignin synthesis remains to be studied in the future.

### 2.8. Expression Analysis by qRT-PCR

**Figure 7:**
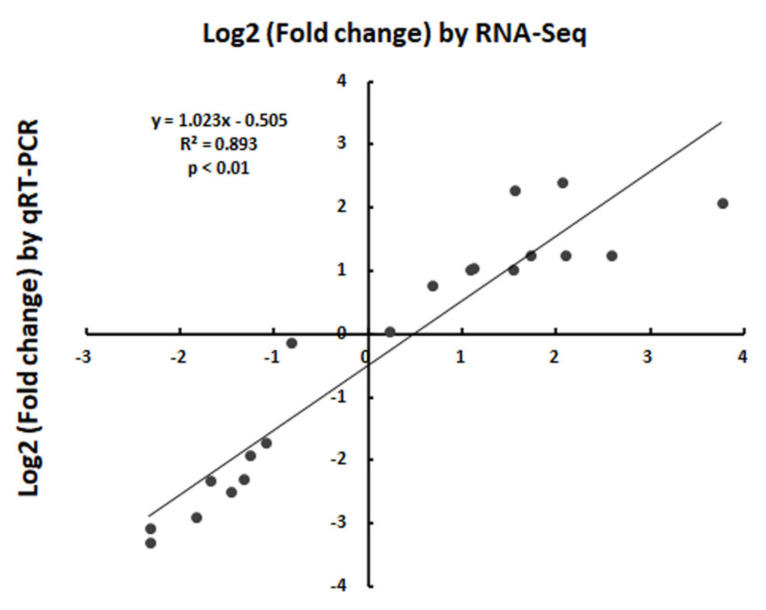
Validation of qRT−PCR. The *Y*-axis indicates the log_2_ (fold change) by RNA−Seq; the *X*-axis indicates the log_2_ (fold change) by qRT−PCR.

To verify the reliability of the transcriptome sequencing data, 10 DEGs related to lignin were chosen for real-time fluorescence quantification (Figure 7 and Table S1). These selected genes included structural genes and TFs. The results of the qRT-PCR show that the expression patterns of these genes were similar to the transcriptome sequencing data, indicating that the transcriptome sequencing results were reliable.

## 3. Discussion

Lignin biosynthesis is a very complex process, which begins with phenylalanine entering the phenylpropanoid metabolic pathway, followed by the lignin-specific synthesis pathway. Finally, three lignin monomers are oxidatively polymerized to three types of lignin by peroxidases in the cell wall [2,20]. In this study, the cell growth of mature leaves was relatively stable, while the cells of juvenile leaves were growing and constructing a cell wall. Thus, it is more important to analyze the expression of lignin biosynthesis genes in juvenile leaves.

The phenylpropanoid metabolic pathway mainly involves the *PAL*, *C4H*, and *4CL* genes. *PAL* is the first rate-limiting enzyme-encoding gene located at the beginning of this pathway and determines the metabolic flow of the entire pathway. As the last key gene, *4CL* regulates the formation of coumaroyl-CoA, caffeoyl-CoA, and feruloyl-CoA, which determines the synthesis of lignin precursors. According to the results of this study, two *4CL2* genes were significantly downregulated in both triploid leaves compared with diploid leaves. Two *PAL1* genes showed no significant difference in juvenile triploid leaves, although they were significantly upregulated in mature triploid leaves compared with diploid leaves. We know that *PAL* is upstream of *4CL*, and that its expression also responds to various outside stresses and plant hormone regulation [21]. Studies have shown that, when genes regulating lignin synthesis change, some other chemical components can also change correspondingly, aiming to ensure the normal physiological metabolism, growth, and development of plants [22,23]. In this study, we found that some flavonoid synthesis genes such as *CHS*, *F3H*, and *FLS* were significantly upregulated in mature triploid leaves. Therefore, we speculated that, during leaf growth and development, *PAL1*, an upstream pathway gene, may lead to more precursors flowing to the branch of flavonoid synthesis, compensating for the reduced lignin accumulation, which might enhance the stress resistance and growth of polyploids. As the final regulatory gene, *4CL2* is widely involved in the synthesis of lignin precursors. Its expression was more in line with the actual lignin synthesis in triploid leaves. Thus, the results indicate that the downregulated expression of key genes in the phenylpropanoid metabolic pathway was one of the reasons leading to the decrease in the lignin content in triploid leaves.

Genes such as *C3H*, *HCT*, *F5H*, *COMT*, and *POD* are the main regulators of the lignin-specific pathway [24,25,26,27]. Among them, *HCT* is key for the synthesis of G and S monomers. *F5H* is necessary for S monomer synthesis. *POD* determines the final oxidative polymerization of monolignols. According to the results of this study, DEGs involved in the lignin-specific pathway were most significantly downregulated in triploids compared with diploids. Among them, *HCT* was downregulated in juvenile triploid leaves. *FAH1* was downregulated in both triploid leaves. A total of four *PODs* were differentially expressed in juvenile triploid leaves, three of which were significantly downregulated. Consequently, these results also indicate that the downregulation of the expression of key genes in this pathway might result in the reduction in lignin content in triploid leaves during leaf growth and development.

Lignin synthesis not only requires the cooperative regulation of structural genes, but also requires the regulation of TFs. Previous studies have shown that a multilevel regulatory network mainly composed of NAC and MYB TFs can regulate lignin biosynthesis [28]. NACs (*VND1–7* and *NST1–3*) are primary switches that regulate secondary cell-wall synthesis. *MYB46* and *MYB83* are secondary switches that can regulate downstream TFs, regulating the expression of secondary cell-wall synthesis genes [28,29]. Among the downstream TFs, *MYB58*, *MYB63*, and *MYB85* can specifically bind to and activate the expression of lignin structural genes [30], whereas *MYB4*, *MYB7*, and *MYB32* are transcriptional inhibitors of lignin synthesis [3]. In this study, the co-expression network between related structural genes and TFs showed that TFs with high degrees of connectivity were mainly MYBs. As an activator of lignin synthesis, *MYB63* was differentially downregulated in triploid leaves compared with diploids, which may lead to decrease lignin accumulation. *MYB4*, a repressor of phenylpropanoid biosynthesis, has been suggested to repress not only the synthesis of monolignols, but also the synthesis of flavonoids [3,31]. In our study, *MYB4* was downregulated in the mature leaves, which may decrease the inhibition of phenylpropanoid pathway and possibly participate in flavonoid synthesis. However, during leaf growth and development, most MYBs have no significant differences, *MYB115* was significantly upregulated in juvenile triploid leaves compared with diploid leaves. *MYB115* shows high homology in amino acid sequence to *Arabidopsis MYB5*, promoting proanthocyanin biosynthesis [32]. Previous studies have shown that certain MYB transcription factors can regulate both flavonoid metabolism and secondary cell-wall formation, such as MYB5-like transcription factors [33,34,35]. *VvMYB5a* and *VvMYB5b* from grapevine positively regulate proanthocyanin and anthocyanin biosynthesis, but negatively affect lignin metabolism [34]. In *Populus tomentosa*, *MYB6*, the homolog of MYB5-like transcription factors, can promote proanthocyanin and anthocyanin biosynthesis and inhibit secondary cell-wall biosynthesis by interacting with *KNAT7*, regulating multiple branches of the phenylpropanoid pathway [35]. In our study, we found that most lignin biosynthesis genes were negatively correlated with flavonoid biosynthesis genes. *MYB115*, as a positive activator of proanthocyanin synthesis, might also participate in the regulation of lignin synthesis. However, further studies are needed to verify whether *MYB115* has a regulatory effect on lignin synthesis.

In this study, our results showed that most genes involved in the regulation of lignin biosynthesis were downregulated during leaf growth and development, leading to a decrease in lignin content in triploid leaves. However, what causes the downregulation of the expression of these genes in triploids? It is known that plant polyploidy can result in cellular giganticity, and poplar triploids are no exception [8,17,36,37]. Zhang [38] showed that phytohormones that including auxin can participate in cell growth, modulating the expression of genes related to cell expansion, and promoting the formation of huge cells. In the study, we analyzed genes related to cell growth, such as *XTHs,* expansins, and plant hormone-related genes, and the results showed that *XTHs* and auxin-related genes may play a major regulatory role in triploid leaves. Their expressions were mostly upregulated, which may lead to the cell growth in triploid leaves. In addition, from the results of this study, most cellulose and hemicellulose biosynthesis genes had no significant difference between triploid leaves and diploids, unlike lignin biosynthesis genes. Only several related genes were differentially downregulated in triploid leaves, indicating that there may be not significant changes or downregulation of chemical component contents between triploid and diploid leaves. However, this needs to be further studied.

From the results of our study, we suppose that polyploids have larger cells, usually accompanied by decreased cell-surface areas per unit volume, which possibly decrease the demand for lignin accumulation. Lignin content is usually reduced in multiple polyploid plants, such as poplar in this study, shrub willow, *Arabidopsis*, rice, and potato [12,13,14,15,16]. In contrast, the cellulose and hemicellulose contents of polyploids showed different trends in different species. For example, the cellulose content increased in triploid shrub willow [13], while it decreased in polyploid *Arabidopsis*, tetraploid rice, and tetraploid potato [14,15,16]. These results indicated that triploids may have a certain mechanism of regulating lignin biosynthesis to adapt their own demands, which are usually achieved by decreasing the expression of lignin structural genes and changing the expression of transcription factors, together regulating lignin biosynthesis. With an increase in chromosome numbers, how do triploids regulate the expression of lignin synthesis genes to adapt to the altered lignin demand due to cell enlargement? In the future, more in-depth and comprehensive studies need to be conducted to explore the molecular regulation mechanism of lignin trait variation in polyploids, such as focusing on woody tissues and studying at the post-transcriptional level. This research provides an important theoretical basis for future studies.

## 4. Materials and Methods

### 4.1. Plant Materials

A synthetic poplar allotriploid (2*n* = 3*x* = 57) and diploid (2*n* = 2*x* = 38) were used as plant materials in this study, which were full-sib progeny induced by *Populus pseudo-simonii* × *P. nigra* ‘Zheyin3#’ and *P. × beijingensis* hybridization [39]. All the materials were planted under 16 h/8 h (day/night) conditions and a relative humidity of 45–70% in the greenhouse of Beijing Forestry University (Beijing, China). The fifth and tenth leaves from the top were collected at the same time when they were 3 months old. Three clones for each ploidy were randomly selected as three biological replicates. After sampling, the materials were immediately frozen in liquid nitrogen and then stored at −80 °C for transcriptome sequencing and other experiments.

### 4.2. Determination of Lignin Content

The lignin content of leaves was determined by ultraviolet spectrophotometry, according to the instructions of a lignin content determination kit (COMINBIO, Suzhou, China). Samples were collected and dried at 105 °C. The dried samples were ground to a fine powder and used to prepare alcohol insoluble residues (AIRs) according to Foster [40]. The pretreated AIRs were used to determine lignin content according to the instructions. Firstly, 5 mg of AIRs were weighed into test tubes, leaving one tube empty for a blank. Then, 1 mL of acetyl bromide solution (25% *v*/*v* acetyl bromide in glacial acetic acid) and 40 µL of perchloric acid were gently added, fully mixed, and heated at 80 °C for 40 min with vortexing every 10 min. After cooling to room temperature, 1 mL of a solution of sodium hydroxide and glacial acetic acid was added to terminate the reaction. Finally, after centrifuging, 40 µL of supernatant was added to 1.96 mL of glacial acetic acid for dilution, and 1 mL of liquid was added to a quartz cuvette to determine absorbance at 280 nm using an ultraviolet spectrophotometer. The lignin content was calculated on the basis of the absorbance at 280 nm, and three biological replicates and three technical replicates were performed.

### 4.3. Histochemical Staining

The leaves, including main veins, were fixed in FAA fixative solution for more than 24 h and used for paraffin sectioning. Firstly, the samples were dehydrated through a gradient of ethanol solutions (70%, 80%, and 100%; 1 h each), followed by ethanol–dimethylbenzene (1:1, *v*/*v*, 30 min) and dimethylbenzene solutions (30 min). Secondly, each sample was transferred to mixtures of paraffin, dimethylbenzene (1:1, *v*/*v*, 60 °C for 6 h), and pure paraffin three times (60 °C for 2 h each time), after which each sample was embedded in a paper cup. Thirdly, the paraffin block was trimmed to a suitable size and sectioned (8 µm) using a paraffin-slicing machine. Lastly, after dewaxing, the paraffin section was stained using 1% safranin O and 0.1% fast green. All the materials were observed and photographed under an Olympus BX51 microscope. The cell areas were measured using ImageJ, and data statistical analysis was conducted using SPSS 20.0.

### 4.4. Transcriptome Sequencing and Mapping

Total RNA was extracted from the samples using TRIzol Reagent Kits (Invitrogen, Carlsbad, CA, USA), followed by using a NanoDrop 2000 bioanalyzer (Thermo Fisher Scientific Inc., Wilmington, DE, USA) to determine the quality. The RNA integrity was verified using 1.5% agarose gels and then was used to construct cDNA libraries using an Ion Total RNA-Seq kit v2. Transcriptome sequencing was performed on the Ion Proton platform (Life Technologies, Carlsbad, CA, USA) by Shanghai Novelbio Biological Technology Co. Ltd., Shanghai, China. The adapter sequences were removed from the raw data. Low-quality reads and reads shorter than 50 bp were filtered out to obtain clean reads using Fast QC [41]. Then, the clean reads were mapped to the genome of *Populus trichocarpa* using MapSplice [42].

### 4.5. Identification of Differentially Expressed Genes and Functional Analysis

The transcript abundance was calculated in reads per kilobase per million mapped reads (RPKM) [43]. Differential expression analysis was performed using DESeq [44]. Genes were deemed to be differentially expressed when they had a fold change (FC) ≥2 and false discovery rate (FDR) <0.05. Differentially expressed genes (DEGs) were annotated by Gene Ontology (GO) and Kyoto Encyclopedia of Genes and Genomes (KEGG) enrichment analysis. The GO enrichment analysis was performed using agriGO (http://bioinfo.cau.edu.cn/agriGO/) (accessed on 10 April 2022) [45]. GO terms with FDR <0.05 were considered significantly enriched terms. DEGs were mapped to the KEGG database (http://www.kegg.jp/) (accessed on 10 April 2022) to identify signaling and metabolic pathways [46]. Pathways were considered to be significantly enriched when *p* < 0.05.

### 4.6. Co-Expression Network Analysis

The RPKM of the genes was used for co-expression analysis. The gene correlation was calculated in terms of the Spearman correlation coefficient using the R package. The screening threshold was |*r*| ≥ 0.85 and *p* < 0.05. A positive value indicated a positive correlation, and a negative value indicated a negative correlation. The co-expression network diagram was visualized using the software Cytoscape 3.9.0, and the connectivity degree of genes was calculated using the same software. The node size was positively correlated with the degree of the connectivity of the genes.

### 4.7. Quantitative Real-Time PCR

Firstly, the total RNA was extracted using RNeasy Plant Mini Kits (Qiagen China, Shanghai, China). Then, cDNA was synthesized using FastQuant RT Kit (with gDNase) (Tiangen Biotech CO., LTD, Beijing, China). The qRT-PCR was accomplished with a SuperReal PreMix Plus (SYBR Green) kit (Tiangen Biotech) according to the manufacturer’s recommendations using an Applied Biosystems 7500 Fast Instrument (AB Ltd., Lincoln, NE, USA). Three technical replicates and three biological replicates were used for each gene. The relative expression of the selected genes was calculated using the 2^−ΔΔCT^ method. The sequences of the primers were designed using Primer3Plus (http://www.primer3plus.com/) (accessed on 10 April 2022). All the primer sequences used in this study are listed in Table S1. Actin (accession number: EF145577) was chosen as the reference gene.

## 5. Conclusions

In summary, our results show that the lignin content in triploid poplar leaves was significantly lower than that in diploid leaves. In the process of triploid lignin biosynthesis, most DEGs were significantly downregulated, which may lead to a decrease in lignin content. At the same time, transcription factors may play an important regulatory role, together resulting in the reduced lignin content. The change in the expression pattern of lignin-related genes in triploids may be a type of adjustment and adaptation to the decreased demand for lignin accumulation.

## Data Availability

The data presented in this study are available in Supplementary Materials.

## Supplementary Materials

The following supporting information can be downloaded at https://www.mdpi.com/article/10.3390/ijms23094603/s1.

## References

[1] Barros J., Serk H., Granlund I., Pesquet E. (2015). The cell biology of lignification in higher plants. Ann. Bot..

[2] Liu Q., Luo L., Zheng L. (2018). Lignins: Biosynthesis and Biological Functions in Plants. Int. J. Mol. Sci..

[3] Yoon J., Choi H., An G. (2015). Roles of lignin biosynthesis and regulatory genes in plant development. J. Integr. Plant Biol..

[4] Li Q., Song J., Peng S., Wang J.P., Qu G.Z., Sederoff R.R., Chiang V.L. (2014). Plant biotechnology for lignocellulosic biofuel production. Plant Biotechnol. J..

[5] Ragauskas A.J., Williams C.K., Davison B.H., Britovsek G., Tschaplinski T., Sørensen B., Green M.A., Lund P., Luque A., MacGill I., Meibom P., Meyer N.I., Patterson W., Pedersen S.L., Rabl A. (2018). The Path Forward for Biofuels and Biomaterials. Renewable Energy.

[6] Marriott P.E., Gómez L.D., McQueen-Mason S.J. (2016). Unlocking the potential of lignocellulosic biomass through plant science. New Phytol..

[7] Warner D.A., Edwards G.E. (1993). Effects of polyploidy on photosynthesis. Photosynth. Res..

[8] Allario T., Brumos J., Colmenero-Flores J.M., Tadeo F., Froelicher Y., Talon M., Navarro L., Ollitrault P., Morillon R. (2011). Large changes in anatomy and physiology between diploid Rangpur lime (*Citrus limonia*) and its autotetraploid are not associated with large changes in leaf gene expression. J. Exp. Bot..

[9] Notsuka K., Tsuru T., Shiraishi M. (2008). Induced Polyploid Grapes via in vitro Chromosome Doubling. J. Jap. Soc. Hortic. Sci..

[10] Yang J., Wang J.Z., Liu Z., Xiong T., Lan H. (2018). Megaspore Chromosome Doubling in Eucalyptus urophylla S.T. Blake Induced by Colchicine Treatment to Produce Triploids. Forests.

[11] Xu J., Jin J., Zhao H., Li K. (2019). Drought stress tolerance analysis of *Populus ussuriensis* clones with different ploidies. J. For. Res..

[12] Yao C.L., Pu J.W. (1998). Timber Characteristics and Pulp Properties of the Triploid of *Populus tomentosa*. J. Beijing For. Uni..

[13] Serapiglia M.J., Gouker F.E., Hart J.F., Unda F., Mansfield S.D., Stipanovic A.J., Smart L.B. (2015). Ploidy Level Affects Important Biomass Traits of Novel Shrub Willow (Salix) Hybrids. Bioenerg. Res..

[14] Corneillie S., de Storme N., van Acker R., Fangel J.U., de Bruyne M., de Rycke R., Geelen D., Willats W.G.T., Vanholme B., Boerjan W. (2019). Polyploidy Affects Plant Growth and Alters Cell Wall Composition. Plant Physiol..

[15] Chen C., Chen Z., Chen J., Huang J., Li H., Sun S., Liu X., Wu A., Wang B. (2020). Profiling of Chemical and Structural Composition of Lignocellulosic Biomasses in Tetraploid Rice Straw. Polymers.

[16] Madadi M., Zhao K., Wang Y., Wang Y., Tang S.W., Xia T., Jin N., Xu Z., Li G., Qi Z. (2021). Modified lignocellulose and rich starch for complete saccharification to maximize bioethanol in distinct polyploidy potato straw. Carbohydr. Polym..

[17] Du K., Liao T., Ren Y., Geng X., Kang X. (2020). Molecular Mechanism of Vegetative Growth Advantage in Allotriploid Populus. Int. J. Mol. Sci..

[18] Liao T., Cheng S., Zhu X., Min Y., Kang X. (2016). Effects of triploid status on growth, photosynthesis, and leaf area in Populus. Trees.

[19] Zhang Q., Xie Z., Zhang R., Xu P., Liu H., Yang H., Doblin M.S., Bacic A., Li L. (2018). Blue Light Regulates Secondary Cell Wall Thickening via MYC2/MYC4 Activation of the NST1-Directed Transcriptional Network in Arabidopsis. Plant Cell.

[20] Baucher M., Monties B., Montagu M.V., Boerjan W. (2010). Biosynthesis and Genetic Engineering of Lignin. Crit. Rev. Plant Sci..

[21] Huang J., Gu M., Lai Z., Fan B., Shi K., Zhou Y.H., Yu J.Q., Chen Z. (2010). Functional analysis of the Arabidopsis PAL gene family in plant growth, development, and response to environmental stress. Plant Physiol..

[22] Voelker S.L., Lachenbruch B., Meinzer F.C., Strauss S.H. (2011). Reduced wood stiffness and strength, and altered stem form, in young antisense 4CL transgenic poplars with reduced lignin contents. New Phytol..

[23] Fornalé S., Capellades M., Encina A., Wang K., Irar S., Lapierre C., Ruel K., Joseleau J.P., Berenguer J., Puigdomènech P. (2012). Altered lignin biosynthesis improves cellulosic bioethanol production in transgenic maize plants down-regulated for cinnamyl alcohol dehydrogenase. Mol. Plant.

[24] Li L., Zhou Y., Cheng X., Sun J., Marita J.M., Ralph J., Chiang V.L. (2003). Combinatorial modification of multiple lignin traits in trees through multigene cotransformation. Proc. Natl. Acad. Sci. USA.

[25] Wagner A., Tobimatsu Y., Phillips L., Flint H., Torr K., Donaldson L., Pears L., Ralph J. (2011). CCoAOMT suppression modifies lignin composition in Pinus radiata. Plant J..

[26] Wang J.P., Chuang L., Loziuk P.L., Chen H., Lin Y.C., Shi R., Qu G.Z., Muddiman D.C., Sederoff R.R., Chiang V.L. (2015). Phosphorylation is an on/off switch for 5-hydroxyconiferaldehyde O-methyltransferase activity in poplar monolignol biosynthesis. Proc. Natl. Acad. Sci. USA.

[27] Wagner A., Tobimatsu Y., Phillips L., Flint H., Geddes B., Lu F., Ralph J. (2015). Syringyl lignin production in conifers: Proof of concept in a Pine tracheary element system. Proc. Natl. Acad. Sci. USA.

[28] Zhong R., Ye Z.H. (2014). Complexity of the transcriptional network controlling secondary wall biosynthesis. Plant Sci..

[29] McCarthy R.L., Zhong R., Ye Z.H. (2009). MYB83 is a direct target of SND1 and acts redundantly with MYB46 in the regulation of secondary cell wall biosynthesis in Arabidopsis. Plant Cell Physiol..

[30] Zhou J., Lee C., Zhong R., Ye Z.H. (2009). MYB58 and MYB63 are transcriptional activators of the lignin biosynthetic pathway during secondary cell wall formation in Arabidopsis. Plant Cell.

[31] Wang X.C., Wu J., Guan M.L., Zhao C.H., Geng P., Zhao Q. (2020). Arabidopsis MYB4 plays dual roles in flavonoid biosynthesis. Plant J..

[32] Wang L., Ran L., Hou Y., Tian Q., Li C., Liu R., Fan D., Luo K. (2017). The transcription factor MYB115 contributes to the regulation of proanthocyanidin biosynthesis and enhances fungal resistance in poplar. New Phytol..

[33] Bhargava A., Mansfield S.D., Hall H.C., Douglas C.J., Ellis B.E. (2010). MYB75 functions in regulation of secondary cell wall formation in the Arabidopsis inflorescence stem. Plant Physiol..

[34] Deluc L., Barrieu F., Marchive C., Lauvergeat V., Decendit A., Richard T., Carde J.P., Mérillon J.M., Hamdi S. (2006). Characterization of a grapevine R2R3-MYB transcription factor that regulates the phenylpropanoid pathway. Plant Physiol..

[35] Wang L., Lu W., Ran L., Dou L., Yao S., Hu J., Fan D., Li C., Luo K. (2019). R2R3-MYB transcription factor MYB6 promotes anthocyanin and proanthocyanidin biosynthesis but inhibits secondary cell wall formation in Populus tomentosa. Plant J..

[36] Yao H., Kato A., Mooney B., Birchler J.A. (2011). Phenotypic and gene expression analyses of a ploidy series of maize inbred Oh43. Plant Mol. Biol..

[37] Ren Y., Jing Y., Kang X.J.P.C.T., Culture O. (2021). In vitro induction of tetraploid and resulting trait variation in *Populus alba* × *Populus glandulosa* clone 84 K. Plant Cell Tissue Organ Cult..

[38] Zhang Y. (2021). The Molecular Mechanism of the Formation of Allopolyploid Giant Cells in *Populus* spp. (*Section Tacamahaca*). Ph.D. Dissertation.

[39] Dong C.B., Suo Y.J., Wang J., Kang X.Y. (2014). Analysis of transmission of heterozygosity by 2n gametes in *Populus* (Salicaceae). Tree Genet. Genomes.

[40] Foster C.E., Martin T.M., Pauly M. (2010). Comprehensive compositional analysis of plant cell walls (*Lignocellulosic biomass*) part I: Lignin. JoVE.

[41] Langmead B., Trapnell C., Pop M., Salzberg S.L. (2009). Ultrafast and memory-efficient alignment of short DNA sequences to the human genome. Genome Biol..

[42] Wang K., Singh D., Zeng Z., Coleman S.J., Huang Y., Savich G.L., He X., Mieczkowski P., Grimm S.A., Perou C.M. (2010). MapSplice: Accurate mapping of RNA-seq reads for splice junction discovery. Nucl. Acids Res..

[43] Mortazavi A., Williams B.A., McCue K., Schaeffer L., Wold B. (2008). Mapping and quantifying mammalian transcriptomes by RNA-Seq. Nat. Methods.

[44] Anders S., Huber W. (2010). Differential expression analysis for sequence count data. Genome Biol..

[45] Ashburner M., Ball C.A., Blake J.A., Botstein D., Butler H., Cherry J.M., Davis A.P., Dolinski K., Dwight S.S., Eppig J.T. (2000). Gene ontology: Tool for the unification of biology. The Gene Ontology Consortium. Nat. Genet..

[46] Tarca A.L., Done A., Georgescu C., Romero R., Khatri P., Draghici S., Voichita C., Amin K. (2013). A systems biology approach for pathway level analysis. Genome Res..

